# Multiple-Feature Extracting Modules Based Leak Mining System Design

**DOI:** 10.1155/2013/704865

**Published:** 2013-12-17

**Authors:** Ying-Chiang Cho, Jen-Yi Pan

**Affiliations:** Department of Electrical Engineering, CCU University, Chia-Yi 62102, Taiwan

## Abstract

Over the years, human dependence on the Internet has increased dramatically. A large amount of information is placed on the Internet and retrieved from it daily, which makes web security in terms of online information a major concern. In recent years, the most problematic issues in web security have been e-mail address leakage and SQL injection attacks. There are many possible causes of information leakage, such as inadequate precautions during the programming process, which lead to the leakage of e-mail addresses entered online or insufficient protection of database information, a loophole that enables malicious users to steal online content. In this paper, we implement a crawler mining system that is equipped with SQL injection vulnerability detection, by means of an algorithm developed for the web crawler. In addition, we analyze portal sites of the governments of various countries or regions in order to investigate the information leaking status of each site. Subsequently, we analyze the database structure and content of each site, using the data collected. Thus, we make use of practical verification in order to focus on information security and privacy through black-box testing.

## 1. Introduction

This paper discusses the issues of e-mail address leakage [1] and SQL injection [2] in online information security. Hackers commonly operate by means of social networks, APT (advanced persistent threat) and spam, and all of these methods begin with the acquisition of the e-mail addresses of their victims [3, 4]. Possible reasons for e-mail addresses being detected are (1) the web programmer not filtering the symbol “@”, the most significant character of an e-mail address, which can be captured by the 24-hour running crawler mining system, and (2) website programmers not being sufficiently rigorous in implementing the “robots.txt” file that prevents crawler programs from accessing the site. Before such automated programs access a page, they determine whether the site contains the robots.txt file that limits their access to specific sites [5]. All normal and well-intentioned programs follow the command of this file [6]; however, the robots.txt is not always enforceable, which means that some spammers and other malicious programs are still able to ignore it [7]. Therefore, it is necessary for certain website programmers to protect confidential online information with a password or an improved program writing style [8, 9].

The Internet environment today is very different from how it was 20 years ago. Firstly, different kinds of automated programs for data collection are continually being developed. Malicious users are able to spread hazardous e-mails after having gathered e-mail address information, accompanied by automatic sending programs, which is a serious information security problem [1, 3, 10]. Secondly, the database that stores the core information for each site requires sufficient protection, and therefore, website programmers need to attempt to prevent malicious pervasive injection attacks by means of improved programming styles [11].

To solve above problems, this study implements a crawler mining system equipped with black-box testing thinking [12–14]. This system consists of three main modules: dynamic scanning module, static mining module, manual operating module, and six functions: data syntax analysis function, hidden page exploring function, multidomain searching on one IP function, specific file searching function, a search engine assistant function, and a website vulnerability updating function. This study analyzes governmental web portals as measured objects, with 24 hours as a unit, where the number of e-mail addresses collected and injectable URLs detected in each is calculated in order to compare the degree of concern around information security in different countries or regions.

This paper proceeds as follows.  Section 2 discusses the previous algorithms.  Section 3 clarifies the system design analysis.  Section 4 presents experiment and result analysis, and Section 5 concludes.

## 2. Previous Algorithms

There are currently several searching strategies commonly used by web crawlers, which are discussed below.

*Algorithm Based on IP Address.* The web crawler obtains a starting IP address, and then proceeds to search the file document of each website included in this IP address one at a time. This method does not take into consideration the URL addresses from pages that lead to other websites. The advantage of this method is the ability to search the pages that cannot be hyperlinked; the disadvantage is that it cannot crawl the entire website. Some modules of our proposed system adopt this strategy. 

*Algorithm Based on Breadth-First Searching.* In the crawling process, after completing the searching of the first level, we shall move on to the next level, and so on, until the searching is completed. In order to cover as many pages as possible, we generally use breadth-first search. Other research has applied breadth-first search to focus the crawler [15, 16]. The main idea behind this method is that there is a large possibility of the starting URL having a theme connection with the pages that are within a certain linking distance [15, 17, 18]. The crawler of the breath-first search gathers all the pages of the starting page and chooses one link from which to take all the pages. Owing to this, the web crawler is capable of speeding up parallel processing. Some modules of our system make use of this strategy. 

*Algorithm Based on Depth-First Searching.* The aim of such an algorithm is to reach the end of searched websites, including those websites that do not contain any hyperlinks. When the hyperlink of a page is chosen, the pages connected to it will undergo a depth-first search (DFS). Before searching the rest of the links, the crawler must completely search this single chain. The crawler will move to the end, along the hyperlink from the page, and then return to another page, continually selecting hyperlinks. When there are no further hyperlinks to choose, the search comes to an end [19, 20]. The advantage of this method is the ability to search through an entire website. The disadvantage is that the crawler may easily become trapped, since the structure of some websites is rather complex and deep. 

*Algorithm Based on Best-First Searching.* This method makes use of the specific webpage analysis algorithm to set a standard value against which the target directed by the URL is evaluated, gathering those URLs that are determined to be above the standard value. The algorithm is limited to the pages whose relation is larger than the standard value set by the webpage analysis algorithm. The disadvantage of this method is that it is likely to ignore many pages during the crawling process, as it is a partial searching method [21]. Therefore, it should be used alongside other application methods in order to achieve optimum performance [22, 23]. 

*Algorithm Based on Reinforcement Learning.* Rennie and McCallum incorporated reinforcement learning into the web crawler's learning process [24]. This method is based on machine learning and is divided into the training phase and the crawling phase. It makes use of reinforcement learning to calculate the future return value, the link for which is provided to form the training module. Thereafter, it predicts the actual future return value by using the training module in the crawling phase, in order to determine the visiting priority of the link. 

*Algorithm Based on the Context Graph.* The algorithm for the web crawler based on the context graph is able to determine the searching direction by means of the *Q* value, but it is incapable of estimating the distance to the target page. Therefore, Diligentim et al. proposed a new searching strategy based on the context graph [22, 25, 26] that estimates the distance to the target page by forming a typical webpage under the context graph. The closer the page, the sooner it is crawled, with the closest being crawled first. 

*Algorithms Based on Content Evaluation or Link Structure Evaluation.* Representative methods based on content evaluation include the Fish-search algorithm [27], the shark-search algorithm [28], the neural network search algorithm, and the web hyperlink analysis algorithm [29–32], while Page Rank [30] and Hits [33] are algorithms based on link structure evaluation. The Page Rank algorithm attempts to determine the importance of a page based on other associated pages of a good quality [34, 35]. The basic idea behind the Hits algorithm is to use the reference link among pages to obtain useful information hidden within the pages through two weight evaluations, Authority and Hub, in order to evaluate the quality of pages [36]. The results obtained from Hits are highly dependent on the chosen starting figures. It is easy to generate theme drift, since the theme authority differs between pages, which aims at different themes. In order to resolve the atmosphere above, the ARC algorithm [37] as well as the Clever algorithm [38] have been developed, which take into consideration the performance of the theme search. 

*Algorithm Based on Classifier-Guided Crawlers.* Although the algorithm based on classifier-guided crawlers is an effective method, there has been little research relating to it [39]. Chakrabarti et al. proposed the focused crawling system [25], which is based on the Naive Bayes classifier. Menczer and Belew presented the Infospiders system [40], which is based on the agent formulae of a genetic algorithm and reinforcement learning. This model is composed of collateral crawls, each of which has its own prediction classifier. Johnson et al. proposed theme crawls based on a support vector machine [41]. 

*Other Algorithms.* In addition to the above, many other web crawler algorithms have been developed. For example, [42] described the case of certain page contents being connected to irrelevant pages. Therefore, an algorithm is needed for crawlers to bypass irrelevant pages in order to reach the related ones. There is also an algorithm that limits the scope of themes that crawlers focus on, such as DNS, link class, IP, or national language. Some modules of our system adopt this strategy.

## 3. System Design

**Figure 1:**
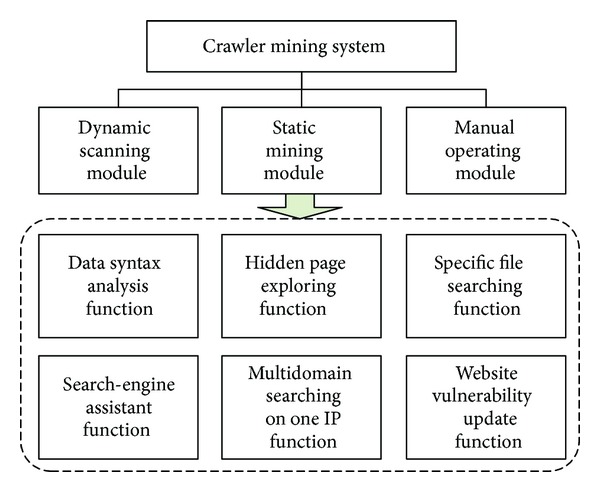
Crawler mining system framework.

**Figure 2:**
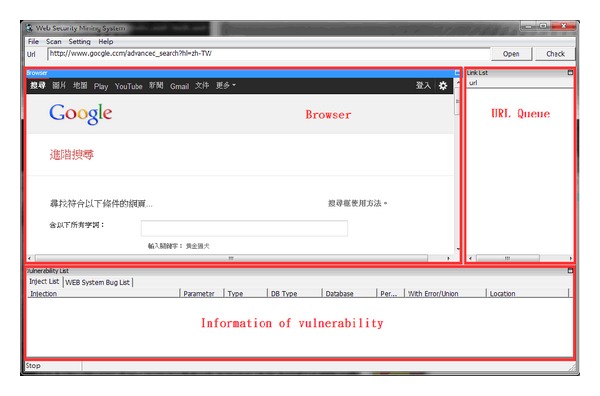
Crawler mining system interface.

This research implements the crawler mining system, which was developed according to the algorithms above, using the JAVA SE7 version with more than 70,000 rows of coding. It can be installed normally in JRE (Java Runtime Environment) on WinXP/Vista/Win7. This system contains three modules and six functions, as shown in Figure 1.  Figure 2 shows the main interface of the system, which is divided into three parts: the browser is in the middle, on the right is the URL queue to be examined, and the list of vulnerable information appears at the bottom.

The crawler mining system is capable of carrying out injections to penetrate databases such as MS-SQL, MySQL, Oracle, PostgreSQL, SQLite, and Access, as well as web languages such as ASP, ASPX, PHP, JSP, and CGI.

### 3.1. Design Principle Analysis

#### 3.1.1. Dynamic Scanning Module

**Figure 3:**
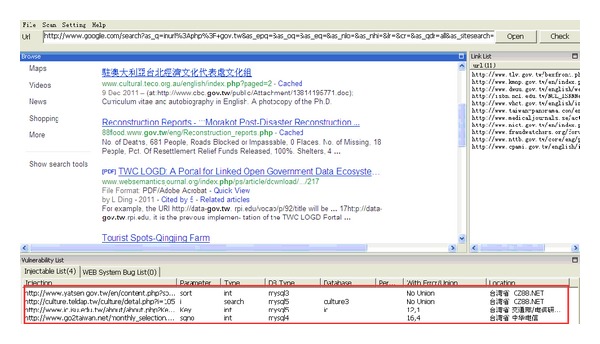
Official websites detection.

**Figure 4:**
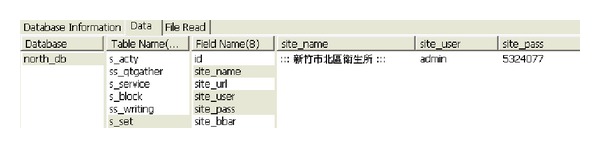
Official website stores database password in plain text.

During the browsing process, the system automatically detects the current browsers one at a time, which makes it easy for the user to be aware of leaks that may occur in the website. Attacks that disclose information from websites can easily take place.  Figure 3 provides a basic example, showing how the crawler mining system searches in Google for official websites. The system will immediately filter all the URLs that appear in the browser. When the system obtains a new URL, it places it into the queue list, and vulnerable URLs are placed into the injection list for further analysis.  Figure 4 shows the results of the further analysis of the vulnerable websites, where the crawler mining system checks the database lists of the websites [43]. The database password for this website can be found as it is saved in plain text, which does not provide adequate security. When malicious attackers obtain this information, they are able to infiltrate this website immediately.

#### 3.1.2. Static Mining Module

**Figure 5:**
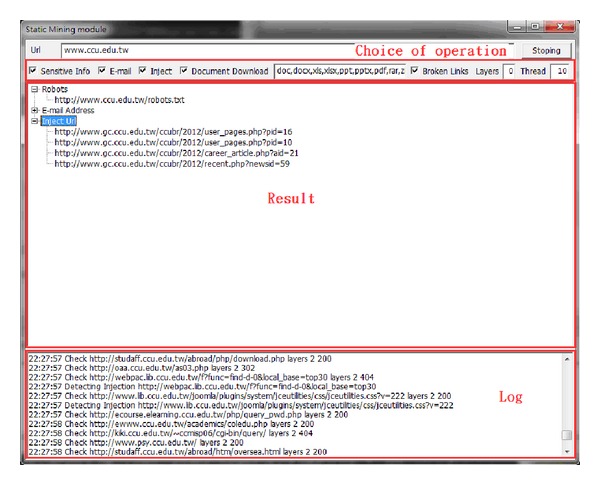
Static mining module.

**Figure 6:**
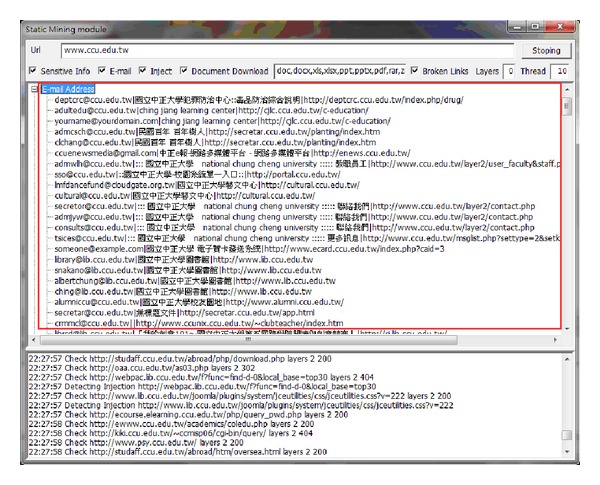
E-mail address leak from the website.

**Figure 7:**
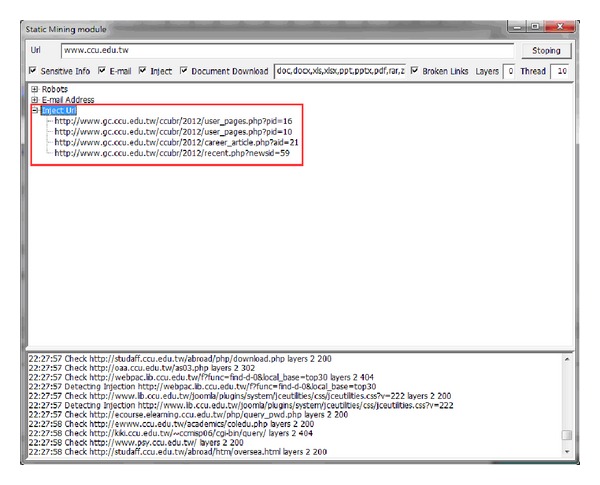
List of SQL injection URLs.

**Figure 8:**
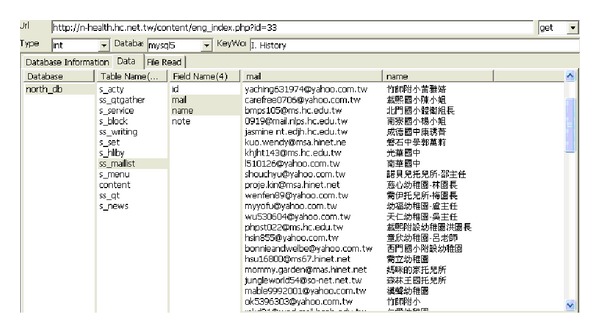
E-mail address leak in the database.

This type of module is used to conduct deep digging in specific websites, with its purpose being classified as static searching. The sensitive information function decides whether it is followed by the robots.txt of the website. Robots.txt is a document coded by ASCII that is stored in the website's root directory and tells the web crawler which content can or cannot be accessed [7]. Other hackers filter the e-mail accounts that are leaked from the website, inspecting risky injection URLs, downloading documents with various filename extensions, or picking up the broken links containing private information.  Figure 5 illustrates the static mining module. The crawler mining system is able to filter the e-mail addresses that are leaked from websites; this process is illustrated in Figure 6. The system is capable of determining injection weaknesses or the major loopholes of the website, as shown in Figure 7. These injection URLs can be used in conjunction with the built-in data programming analysis function in order to inject into the database of the website to obtain the usernames and e-mail accounts stored within it.  Figure 8 demonstrates that, when malicious users obtain this information, they may perform various malicious attacks, such as spreading e-mails with viruses, conducting social engineering attacks to gain users' passwords, or obtaining personal information by infiltrating the database of the website [3, 10]. The more injection URLs that are detected by the crawler mining system, the more logical programming errors this website contains, leading to the invasion risk being relatively high.

#### 3.1.3. Manual Operating Module

All of the functions that constitute the dynamic scanning module and the static mining module are automatic; however, human logic is the most important factor when it comes to web security [44]. The various functions that can be conducted by humans form the manual operation module.

### 3.2. Function Analysis

#### 3.2.1. Data Syntax Analysis Function

Based on SQL injection [45, 46], we use the system to conduct an examination of the database of a website [11]. When an issue in the programming of the website causes the system to be incapable of determining whether the figures injected are attached to SQL orders, these attached orders will run normally, since the database does not detect the injection. In this way, illegal users are able to obtain database information, which causes serious problems in information security [47]. Users may then be hacked because their information is leaked, the website structure is invaded illegally, or the administrator's account is altered to gain higher authority in order for malicious links or cross-site scripting to be added to the website [48]. When the system conducts the examination, the first step is to search for the injection point, to judge whether the website contains any harmful designs. Thereafter, a syntax dictionary is added to this system through the URL in order to block information and automatically judge from the feedback of the website whether anything will be achieved by conducting digging. The following shows the basic syntax injection:

- http://www.xxxx.gov.tw/fwdt/fw/list_show.asp?cateid=457 and 1=1
- http://wj.xxxx.gov.tw/xxqk/InfoPublic/PublicView. asp?id=60 and user=0 and '8'='8'.

The information leaks are evaluated according to the string of the syntax dictionary. When the system indicates that there is value in continuing digging, the type of database this website makes use of will be determined by means of the different functions defined by the database. For example, MS-SQL and MySQL use len( ) to calculate objective length, while Oracle does this according to length. Therefore, when using a len(“s”) = 1 for examination, normal feedback is obtained, which means the target website uses a MS-SOL or MySQL database; however, when this is not the case, the website may use Oracle. Additional functions defined by the database are also able to perform these evaluations. Once the database type has been established, the form fields of the database are analyzed, and finally, the figures and information of the database are obtained [43, 45, 46].

#### 3.2.2. Hidden Page Exploring Function

There is some website information that is not easily detected or obtained by crawlers [49], such as paid journal articles saved in libraries or membership accounts of commercial websites, and this content is known as the Deep Web [50]. Some website designers believe that the website administration page not being obviously shown prevents easy browsing by users, and thus provides information security. However, the administration page may still be found by using specific filter methods. This system employs such methods to conduct hidden page exploring for examination and analysis of information. The first method is that of using syntax dictionary to explore the administration page according to the page naming rules that are used most, while the second one is exhaustive searching, using characters set up by users to perform permutation combinations.

**Figure 9:**
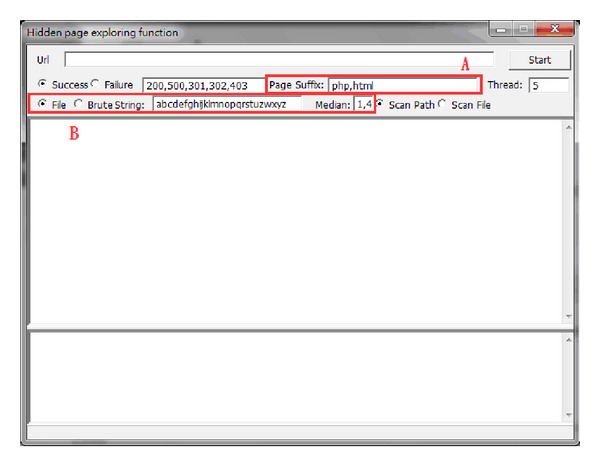
Interface for hidden page scanning.

Figure 9 shows the interface for the scanning of hidden pages. Area A is an addition to the interface for entering a web language, as there is an ever-increasing amount of web languages, such as HTML, ASP, PHP, CGI, and JSP, being used. In area B, when searching for hidden pages, the page name defined by the syntax dictionary is selected for scanning or exhaustive searching, Exhaustive searching examines the given characters and length one at a time; therefore, it takes more time, but has a wider coverage. The page names defined in the syntax dictionary can be added or deleted according to the naming rules that are used the most at any time.

**Figure 10:**
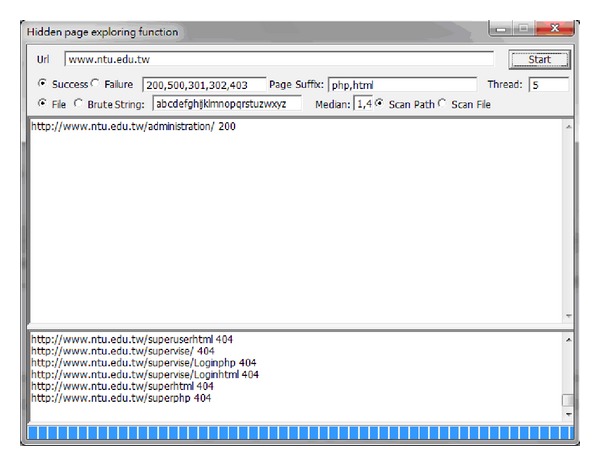
Category page.

The category page for the National Taiwan University website is provided as an example in Figure 10. The administration category page link can be obtained using the hidden page exploring function, by scanning the built-in syntax dictionary. In this example, if the administration interface is not adequately protected, it can easily be attacked by malicious users.

Many modern websites request an account name and password for authority control to be carried out. The proposed system contains a function aimed at the Deep Web that provides enterprises or persons with a feature for testing whether any password used is weak.

**Figure 11:**
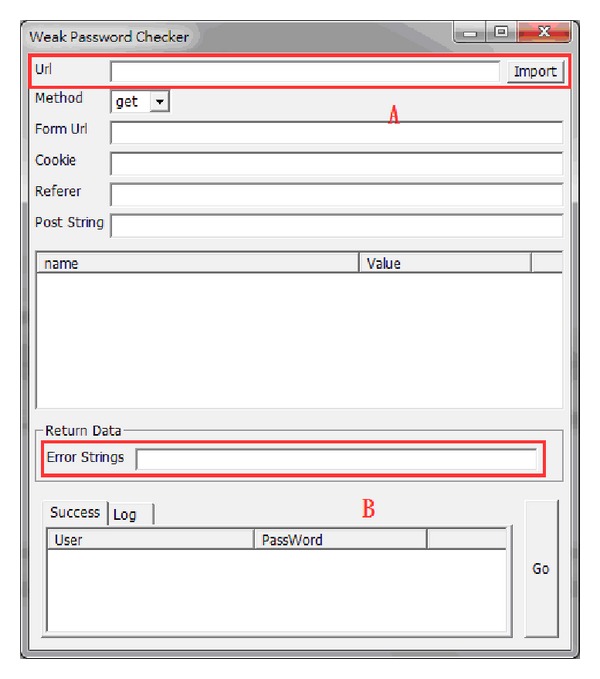
Interface for detecting a weak password.

The URL provided in area A will be analyzed according to the variables and cookies related to input on the webpage. When incorrect account names and passwords are entered into area B, the webpage will show the corresponding string, which can be tested using the given ID/PWD dictionary. If a password in the database is automatically detected as being vulnerable to web crawlers logging on, we shall inform the personnel to change the password in order to protect personal information.  Figure 11 shows the interface for detecting a weak password.

#### 3.2.3. Multidomain Searching on One IP Function

**Figure 12:**
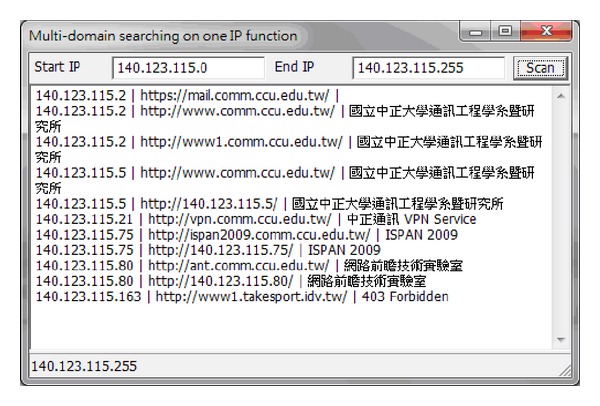
Multidomain searching on one IP function.

Multidomain searching on one IP function built into the web crawler system can be used to explore all the websites in a particular network segment. However, we must know which pages are under service and we must be able to avoid turning on more than two website services from certain servers or IPs; that is, when the TCP PORT is turned on it will not necessarily be in port 80. It can be seen in Figure 12 that there are 11 websites in this network segment in total, with their respective domain names being shown. There are three websites with the IP address 140.123.115.

#### 3.2.4. Specific File Searching Function

In this study, we conduct a DFS on file types of which there are rich resources on the internet. If we wish to search for the file types of PDF, MS-Word, or MS-Excel, for example, we can use the specific file searching function to limit the scope. We are then able to conduct resources searching through the preferred need set-up in order to find the document files that are associated with the preferred conditions.

#### 3.2.5. Search Engine Assistant Function

**Figure 13:**
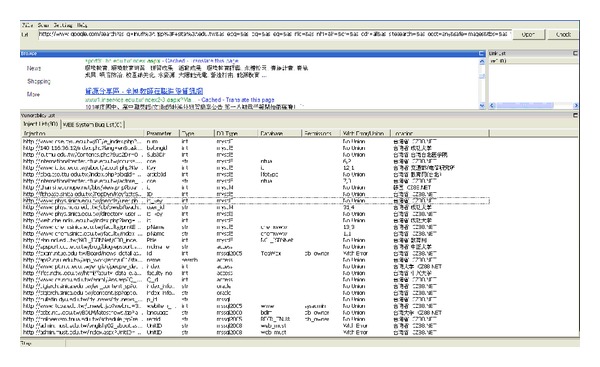
Possibility of leaking in databases.

Modern search engines, such as Google, YAHOO, Bing, and Baidu, are extremely powerful and convenient. We are therefore able to test the government-related websites in Taiwan by means of keyword searching and this system's dynamic scanning module. We do this by entering the keywords “inurl:php? site:gov.tw XX events” in Google to find out XX events relating to recently published material on government-related websites in Taiwan. In this way, we are able to conduct scanning of a specific issue or topic. The question is how should we order the keywords in the Google search engine? We provide an example. We enter the words “Inurl:.php?*|*.asp?*|*.jsp?*|*.apsx?site:edu.tw” into the Google search engine, because we wish to focus on dynamic-type pages for examination, such as PHP, ASP, JSP, or ASPX. In our experiment, we examined 800 samples of data from academic webpage databases of Taiwan; therefore, 200 data items for each type of page. Of the 800 data samples, 76 showed a high possibility of leaking occurring, with 31 being found in PHP; 26 in ASP; 12 in ASPX, and 7 in JSP. These figures are displayed in Figure 13.

**Figure 14:**
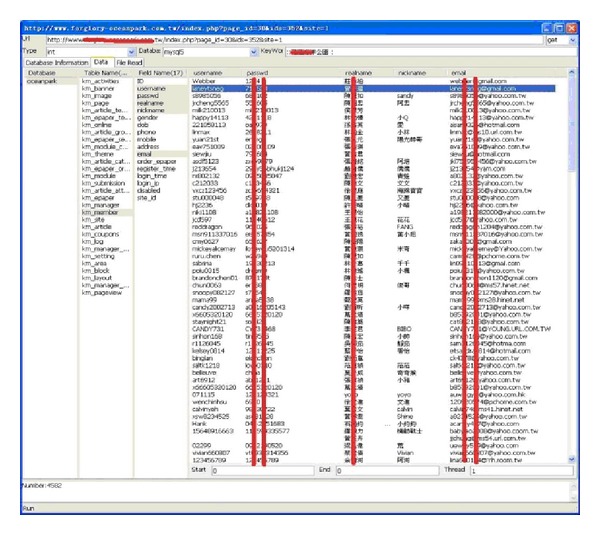
Account names and passwords of a database, stored using plain text.

Using the information of the injection URL, we can apply the data syntax analysis function in our web crawler system, as well as the SQL injection technique, in order to obtain the database contents. Within the Taiwan academic network, 5% of websites still store the account names and passwords of their databases using plain text, as shown in Figure 14. Thus, users' account names and passwords combined are likely to be obtained due to flaws in the design logic of the database.

#### 3.2.6. Website Vulnerability Updating Function

New leaks on the internet are researched by experts regularly. We use the loophole published in the Exploit Database [51] as an example and convert this latest loophole into the forms that are supported by our web crawler system. The forms we apply are shown in Algorithm 1. Note that we ignore the encryption algorithm section.

We are able to verify this loophole example by means of the instantaneous updating of the loophole database. We do not rely on the injection technique alone, as this is very similar to instantaneously updating virus codes.

## 4. Experiment and Result Analysis

In our experiment, we were able to infer the degree of importance that every country places on website security by investigating the level of security implemented in the portal websites. The experimental hardware used were Intel Core I5-2500 CPU, with 8 GB of RAM, using the Microsoft Windows 7 operating system. The experiment time was 24 hours for each county. The figures given in the experimental results were obtained by gathering the follow-up statistics.

### 4.1. Assessment of Government Portal Websites of 24 Countries or Regions

We selected 24 countries or regions to conduct experiments on using the online crawler mining system, by filtering the e-mail addresses and URLs that were leaked by SQL injections into the countries' or regions' government portal websites, as shown in Table 1.

**Figure 15:**
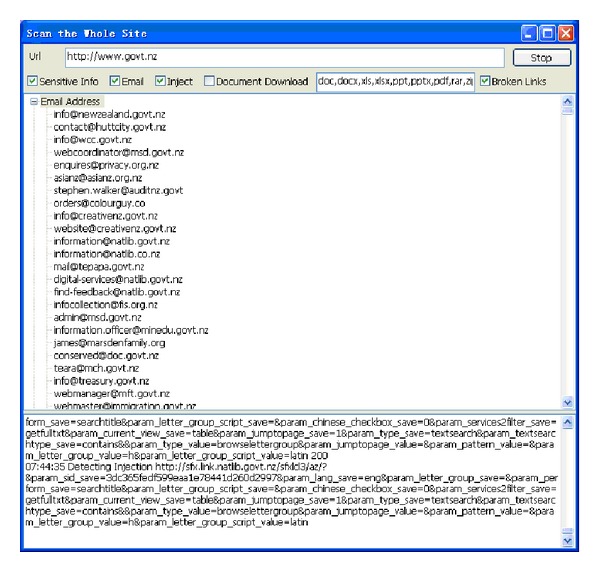
Leaked e-mail addresses in portal website of New Zealand.

**Figure 16:**
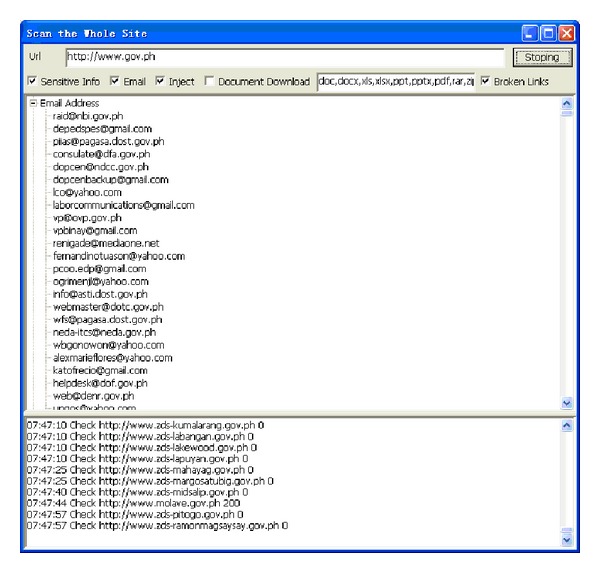
Leaked e-mail addresses in portal website of Philippines.

**Figure 17:**
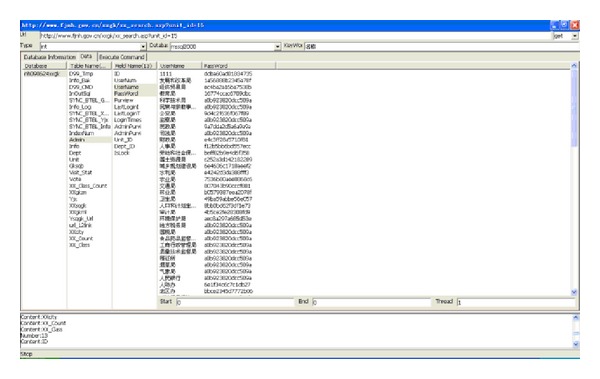
Content of the database obtained from URLs.

Among these 24 countries or regions, the highest levels of protection in the portal websites were found to be those of Germany, Singapore, Italy, Finland, Japan, and India, as no e-mail addresses or URLs were obtained from the injections. The lowest degree of security was found in the government portal websites of New Zealand and the Philippines, with the number of leaks in these being 8972 and 2352, respectively, as shown in Figures 15 and 16. The largest number of URLs obtained from injections was in the government portal of PRC. Although Canada's figure is the highest, these results were obtained from the same database, with only 5 to 10 websites being weak. Furthermore, the crawler mining system analyzed the URLs obtained from the injections, revealing the usernames and passwords stored inside the database, as displayed in Figure 17. When it is possible to dig or permeate the database, malicious users will drill deeply into the system in order to carry out serial malicious attacks.

### 4.2. Comparison of Permanent Memberships of the United Nations Security Council

We analyzed the permanent membership of the United Nations Security Council in 24 countries or regions, as shown in Table 2. The most severe case of leaked e-mail addresses and URLs obtained was in PRC, followed by Russia, while France was found to have the best results. Since the United Nations Security Council is responsible for resolutions within the UN, any opinions raised among factions may affect the outcome of the resolution of an important issue. Therefore, without sufficient information security, hackers may obtain significant information through attacks, which could potentially lead to the manipulation of information, and therefore major problems, within the UN.

## 5. Conclusion

Our experimental results indicate that the most severe leakage problems in government portal websites occur in PRC, with 121 injections, which means that there is a weakness in the website programming, making it relatively easy for many malicious hackers to obtain information. The lowest number of leaks was found in the government portal websites of India, Italy, and Finland, meaning that, in general, these sites are less likely to be attacked. Furthermore, the government portal websites of Germany, Singapore, and Japan could not even be tested or analyzed, which means that they are completely protected against any attacks by the online crawler mining system. It is thus clear that the portal websites of these countries or regions are developed with a much higher level of information security.

On December 21, 2011, a series of leakages of user information occurred in various famous websites in Mainland China, affecting 50 million online users, and was allegedly the largest known online information leakage incident. It is a known fact that no online information can be completely secure; however, research in this area has established four major aspects that should be taken into account in order to improve the protection of online information [2, 52], as follows. (1) Website contents that prohibit the research of reptile programs should be regulated. (2) During the programming of websites, it is necessary to filter the input values and limit the variable modes. (3) Unnecessary extensive processes for database management should be removed, such as xp_cmdshell and xp_regaddmultistring. Passwords stored in the database must be encoded, but not using plain text. (4) A good writing style of robot.txt and network management mechanisms should be implemented. In dealing with attacks, forged files and categories may be added into the robot.txt, programming annotation or error websites. Such mechanism may reveal the intention from attackers or malicious software, which is considered as a Honeypot approach.

SQL injection is not a new technique, and various methods for preventing leakage in databases have been extensively studied [53, 54]. However, over the past decade, this issue has become a major concern in the field of information security. The habits of front users and the concepts of backward development are the main reasons for this concern [11, 55]. We will continue to improve the functions of our system, from an online reptile angle, by comparing our research with that of related fields [47, 48, 56–60], in order to develop a more powerful system.

With the increasing use of computers and the rapid development of the internet, society is experiencing fast-changing and different lifestyles. Information security has become a major concern as easily available online information continues to grow. Hence, measures must be taken for improving information security. Only under more secure conditions will we be able to fully enjoy the convenience of online information, which is necessary for living in the information age and being capable of facing greater challenges.

## Figures and Tables

**Algorithm 1:**
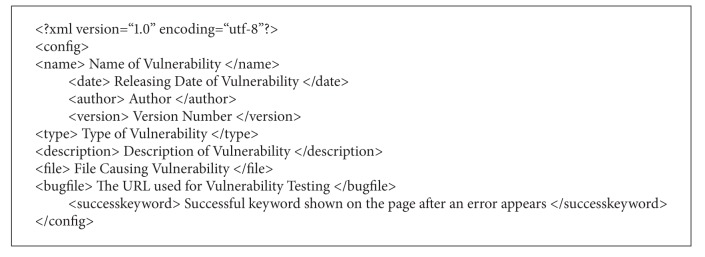


**Table 1:** Testing results of 24 countries or regions.

Country or region	Government portal website address	Number of leaked e-mail addresses	Number of SQL injections
Canada	http://www.canada.gc.ca	1054	1295
PRC	http://www.gov.cn	3287	121
Germany	http://www.ezilon.com/regional/germany/government	0	0
UK	http://www.direct.gov.uk/en/index.htm	1177	0
US	http://www.usa.gov	748	0
Taiwan	http://www.gov.tw	779	15
Singapore	http://www.gov.sg	0	0
Japan	http://www.e-gov.go.jp	0	0
Philippines	http://www.gov.ph	2352	6
France	http://www.premier-ministre.gouv.fr/en	31	0
New Zealand	http://www.govt.nz	8962	3
Australia	http://www.australia.gov.au	1727	0
South Korea	http://www.korea.go.kr	1	0
India	http://india.gov.in	0	0
Argentina	http://www.argentina.gob.ar	217	0
Brazil	http://www.brasil.gov.br	511	0
Portugal	http://www.portugal.gov.pt/pt.aspx	260	0
Spain	http://www.lamoncloa.gob.es/PaginaNoEncontrada.html	1032	0
Italy	http://www.italia.gov.it/itagov2	0	0
Norway	http://www.norway.no	1683	0
Iceland	http://www.stjornarrad.is	4	0
Finland	http://government.fi/etusivu/en.jsp	0	0
Sweden	http://www.sweden.gov.se	3	0
Russia	http://www.gov.ru	3485	12

**Table 2:** Statistics of permanent membership of the United Nations Security Council.

United Nations Security Council	Number of e-mail addresses leaked	Number of SQL injection URLs obtained
US	1776	0
PRC	3287	121
Russia	3485	12
UK	1267	0
France	31	0
